# Breakthrough Pulmonary Nocardiosis Due to *Nocardia cyriacigeorgica* in a Lung Transplant Recipient in Brazil: A Case Report Highlighting Management Without Susceptibility Testing

**DOI:** 10.1155/crdi/2026205

**Published:** 2026-06-12

**Authors:** Caian L. Vinhaes, M. Gabriela Gallegos, Joana R. Deheinzelin, Priscila Leon, Bruno B. Andrade, Silvia Vidal Campos

**Affiliations:** ^1^ Clinical Research Laboratory on Mycobacteria (LAPCLIN-TB), Evandro Chagas National Institute of Infectious Diseases, Oswaldo Cruz Foundation, Rio de Janeiro, Brazil, fiocruz.br; ^2^ Biological Immunology and Resistance Dynamics (BIRD), Multinational Organization Network Sponsoring Translational and Epidemiological Research (MONSTER) Initiative, Salvador, Brazil; ^3^ Infectious Diseases Department, Clinical Hospital of the University of São Paulo, São Paulo, Brazil; ^4^ Pulmonary Division, Heart Institute (InCor), University of São Paulo, São Paulo, Brazil, usp.br; ^5^ Division of Infectious Diseases, Department of Medicine, Johns Hopkins University, Baltimore, Maryland, USA, jhu.edu; ^6^ Department of International Health, Bloomberg School of Public Health, Johns Hopkins University, Baltimore, Maryland, USA, jhu.edu

**Keywords:** lung transplantation, *nocardia*, opportunistic infection

## Abstract

**Background:**

*Nocardia* infection is an emerging concern in solid organ recipients due to prolonged immunosuppression.

**Case Presentation:**

We report the case of a 45‐year‐old woman with a history of cystic fibrosis who underwent bilateral lung transplantation. Eight years posttransplant, she developed progressive respiratory symptoms. Imaging revealed cavitary nodular lesions, and *Nocardia cyriacigeorgica* was isolated from bronchoalveolar lavage and sputum cultures. Treatment with meropenem and trimethoprim‐sulfamethoxazole led to clinical and radiological improvement.

**Conclusion:**

This case highlights the diagnostic challenges of *Nocardia* infections, the importance of microbiological interpretation, and the potential for late‐onset disease in transplant recipients despite prophylaxis. Awareness of *Nocardia* as an opportunistic pathogen is crucial for timely diagnosis and management. Our findings underscore the need for continued vigilance in solid organ transplant recipients, particularly in cases of persistent pulmonary infections and high immunosuppressive therapy.

## 1. Introduction

The advent of effective immunosuppressive therapy after solid organ transplantation (SOT) was one of most important strategies to improve the graft function and survival. However, the use of these agents leads to impairment of the immune response against ubiquitous organism, making the host immunocompromised and increasing the risk of infections due to atypical or opportunistic pathogens. *Nocardia* is a ubiquitous pathogen, and a well‐recognized opportunistic pathogen in immunocompromised hosts. The incidence of nocardial infection after SOT ranges from 0.6% to 2.7% [1] and varies according to the transplanted organ. As it is acquired via environmental inhalation, lung transplant recipients are a population at risk for this infection.

Over 40 species are recognized with potential to cause human infections [2]. A recent study conducted with data from the United States showed *N. farcinica* as the most common *Nocardia* species in infection after SOT, followed by *N. cyriacigeorgica* and *N. nova* [1]. However, robust data on molecular identification of *Nocardia* in Brazil is limited, and the Brazilian Committee on Antimicrobial Susceptibility Testing does not have established cutoff points for the sensitivity test of *Nocardia* species. Nonetheless, adequate identification is crucial to conduct clinical treatment, according to the susceptibility peculiarities that could diverge according to the species [3].

Here, we report a case of *Nocardia* pneumonia after a bilateral lung transplantation due to cystic fibrosis. To our knowledge, this is the first case of *Nocardia* infection in our service, after 21 years in lung transplantation, and one of the first reports of *Nocardia* infection after SOL in Brazil. Our case highlights the clinical complexity to diagnosis and the relevance of the pathogen in a growing population of transplanted hosts receiving trimethoprim‐sulfamethoxazole prophylaxis. This case report was prepared in accordance with the CARE guidelines, and the completed CARE checklist is provided as supporting information (CARE-checklist-English).

## 2. Case Presentation

A 45‐year‐old woman underwent bilateral lung transplantation 8 years before presentation due to cystic fibrosis. Microbiological history revealed chronic colonization by *Pseudomonas aeruginosa* pretransplantation. Maintenance immunosuppressive regimen at admission included tacrolimus (7 mg daily), mycophenolate (2 g daily), and prednisone (5 mg daily). Additionally, she had trimethoprim‐sulfamethoxazole (TMP‐SMX) 80 mg/400 mg daily for pneumocystis prophylaxis and azithromycin 500 mg three times a week for chronic allograft dysfunction (CLAD). Posttransplant, she experienced three episodes of acute rejection, the last in 2018, all treated with corticosteroid pulse therapy.

Eight years after transplantation, she presented to the emergency department with a 5‐day history of dry cough which worsened into a productive cough 2 days prior, associated with rhinorrhea, nasal congestion, frontal headache, and dyspnea. She was discharged with levofloxacin 750 mg daily for 10 days, and increased prednisone dose to 20 mg.

**FIGURE 1:**
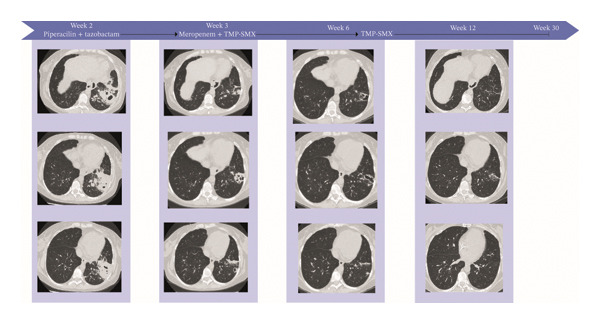
Timeline of the chest‐CT evolution and antimicrobial treatment. Weeks reflect time from initial presentation.

Two weeks later, she returned to the emergency department due to persistent symptoms, reporting worsening cough and new‐onset thoracic pain. Vital signs showed an oxygen saturation of 90% and a heart rate of 103 bpm. Physical examination revealed bibasilar crackles. A chest X‐ray demonstrated new consolidations, and laboratory findings included an elevated leukocyte count (19,200 cells/mm^3^, reference value 4000–11000 cells/mm^3^) and C‐reactive protein (CRP) of 173 mg/L (reference value < 5 mg/L). Given these findings, she was admitted for further evaluation and treatment. A chest CT showed diffusely distributed nodular opacities up to 19 mm diameter, some exhibiting a halo sign, along with a cavitary consolidation in the basal anteromedial and lateral segments of the left lower lobe (Figure 1). Empiric therapy with piperacillin‐tazobactam (4.5 g every 6 h) was initiated.

Bronchoscopy with bronchoalveolar lavage (BAL) was performed. The airways appeared intact bilaterally, with no signs of ischemia, necrosis, dehiscence, or malacia. However, purulent secretions were present throughout the bronchial tree. Molecular testing for *M. tuberculosis*, smear microscopy, and mycobacterial cultures were negative. Fungal culture yielded *Candida albicans*. Aerobic bacterial culture was positive for *Pseudomonas aeruginosa* > 1,000,000 colony‐forming units (CFU), susceptible to all antipseudomonal antibiotics, including piperacillin‐tazobactam. Additionally, *Nocardia cyriacigeorgica* (> 1,000,000 CFU) was isolated from BAL fluid, which was also detected in a subsequent sputum culture.

On Day 10 of piperacillin‐tazobactam therapy, the patient remained symptomatic with persistent dry cough. Repeat laboratory tests showed a leukocyte count of 11,000/mm^3^ and CRP of 2.7 mg/L. A follow‐up chest CT demonstrated persistent irregular parenchymal opacities with a nodular configuration, peribronchovascular distribution, and subpleural involvement, predominantly in the upper lung fields. Endoluminal secretions were noted in some bronchi, along with bronchiectasis in the upper segment of the left lower lobe. Additionally, a small cavitated nodule with thick walls measuring 1.3 cm was identified in the upper lingular segment (Figure 1).

At this moment, the antimicrobial regimen was adjusted to meropenem (3 g daily) and TMP‐SMX (trimethoprim 15 mg/kg daily) after culture results. The patient showed significant clinical improvement with resolution of the cough. Brain CT ruled out central nervous system involvement.

After 19 days of meropenem and TMP‐SMX, a follow‐up chest CT revealed regression of the cavitary lesion in the anterior segment of the left upper lobe, now appearing as an irregular nodular area. There was also a reduction in the size of the previously noted nodule in the same lobe and improvement in peribronchial inflammatory changes in the left lower lobe. Meropenem was continued for three weeks, and next the patient was discharged with TMP‐SMX (10 mg/kg/day) as secondary prophylaxis.

A follow‐up chest CT performed a month after meropenem and TMP‐SMX therapy (Figure 1) confirmed complete resolution of the cavitary lesion in the anteromedial basal segment of the left lower lobe and reduction in scattered nodular opacities in the left lung, indicating radiological improvement. The treatment finished on 6 months later with satisfactory clinical recovery without recurrence of infection.

## 3. Discussion

The improvement of immunosuppression strategies after SOT is crucial to graft survival, ensuring the function and consequently, the wellbeing of the host. However, the state of immunosuppression leads to increased risk of opportunistic infections and challenges in diagnosis and management of unusual infections. In this scenario, *Nocardia* infection emerges as a relevant pathogen in lung recipients.

Data on the epidemiology of nocardiosis in Brazil remain limited, and the true burden of disease is likely underestimated. Available evidence suggests that nocardiosis is a rare but potentially severe infection, predominantly affecting immunocompromised hosts, including transplant recipients and individuals with advanced HIV infection. Brazilian case series are small and heterogeneous, often reflecting single‐center experiences, which limits generalizability. In one report of 22 cases, high mortality rates were observed, particularly among patients with disseminated disease, underscoring the clinical relevance of this infection in vulnerable populations [4].

A retrospective cohort study (1991–2007) including 577 lung recipients reported *Nocardia* infection in 1.9% (11 participants), with a mean onset of 14.3 months posttransplantation [5]. Another retrospective cohort including 473 lung recipients found an incidence of 2.1% (10 patients), with a median onset of 34.1 months after transplantation [6]. A more recent single‐center case‐control study (2012–2018) reported a higher incidence of 3.4% (20/586), with infection occurring at a median of 9.4 months posttransplant [7]. The increased incidence in the more recent study may reflect improved clinical recognition and diagnostic capabilities in transplant centers. Our patient developed symptoms 95 months posttransplant, approximately 8 years, significantly later than reported in the literature, underscoring the risk of *Nocardia* infection in long‐term graft recipients.

The diagnosis and management of pulmonary infection remain challenging [5]. The disease typically presents with nonspecific respiratory symptoms, requiring additional diagnostic tests. However, clinical suspicion remains crucial to guide the investigative steps. As in our case, the manifestation of the disease in lungs is usually a cavitary nodular lesion [8]. Although culture remains the gold standard for diagnosis, clinical interpretation can be complex, often delaying treatment. Distinguishing between colonization and active infection is critical. At first, this raised doubts for our management. However, the isolation of *Nocardia cyriacigeorgica* in bronchoalveolar lavage and sputum, in the presence of a cavitary lung lesion on chest CT, was considered clinically significant and prompted targeted antimicrobial therapy. In lung transplant recipients, the recovery of *Nocardia* from respiratory samples is generally regarded as indicative of active infection rather than colonization, particularly in the presence of compatible radiologic findings Reference [9].

The role of TMP‐SMX prophylaxis in preventing *Nocardia* infection in SOT remains uncertain. Some studies suggest protective effects, particularly with use in the 12 weeks preceding diagnosis [7]. A systematic review and meta‐analysis including 260 SOT recipients reinforced this protective role [10]. However, breakthrough infections have been documented [5, 6], potentially linked to suboptimal prophylactic dosing aimed primarily at *Pneumocystis jirovecii* [11]. Notably, all reported breakthrough infections remained susceptible to TMP‐SMX. Unfortunately, we did not have antimicrobial susceptibility testing due to the absence of established cutoff point in the Brazilian Committee on Antimicrobial Susceptibility Testing.

## 4. Conclusion

This report highlights the clinical relevance of nocardiosis in lung transplant recipients, emphasizing its potential to occur even many years after transplantation. It underscores the diagnostic challenges, including microbiological interpretation and differentiation from colonization, as well as the complexities of treatment in settings with limited access to susceptibility testing. Clinicians should maintain a high index of suspicion for *Nocardia* in immunocompromised patients, particularly when radiologic findings are suggestive, as early recognition and appropriate therapy are critical to improving outcomes.

## Author Contributions

Caian L. Vinhaes, M. Gabriela Gallegos, Joana R. Deheinzelin, and Priscila Leon wrote the main manuscript text. Caian L. Vinhaes prepared figures.

Bruno B. Andrade and Silvia Vidal Campos review the manuscript.

## Funding

No funding was received for this research.

## Consent

Written informed consent was obtained from the patient for publication of this case report and any accompanying images. Identifying information has been anonymized to protect patient privacy.

## Conflicts of Interest

The authors declare no conflicts of interest.

## Supporting Information

Additional supporting information can be found online in the Supporting Information section.

## Supporting information


**Supporting Information** This case report was prepared in accordance with the CARE reporting guideline, and the completed CARE checklist is provided as Supporting Information (CARE‐checklist‐English).

## Data Availability

Data sharing is not applicable to this article as no datasets were generated or analyzed during the current study.
